# An Open-source Toolbox for Analysing and Processing PhysioNet Databases in MATLAB and Octave

**DOI:** 10.5334/jors.bi

**Published:** 2014-09-24

**Authors:** Ikaro Silva, George B. Moody

**Affiliations:** 1 Laboratory for Computational Physiology/PhysioNet, Institute for Medical Engineering and Science, Harvard-MIT Program in Health Sciences and Technology, Massachusetts Institute of Technology, USA

**Keywords:** ECG, QRS, EEG, Arrhythmia, PhysioNet, Biosignal processing, Biomedical, Database, Octave, MATLAB, Physiological Signals

## Abstract

The WaveForm DataBase (WFDB) Toolbox for MATLAB/Octave enables integrated access to PhysioNet’s software and databases. Using the WFDB Toolbox for MATLAB/Octave, users have access to over 50 physiological databases in PhysioNet. The toolbox provides access over 4 TB of biomedical signals including ECG, EEG, EMG, and PLETH. Additionally, most signals are accompanied by metadata such as medical annotations of clinical events: arrhythmias, sleep stages, seizures, hypotensive episodes, etc. Users of this toolbox should easily be able to reproduce, validate, and compare results published based on PhysioNet’s software and databases.

## (1) Overview

### Introduction

**Figure 1:**
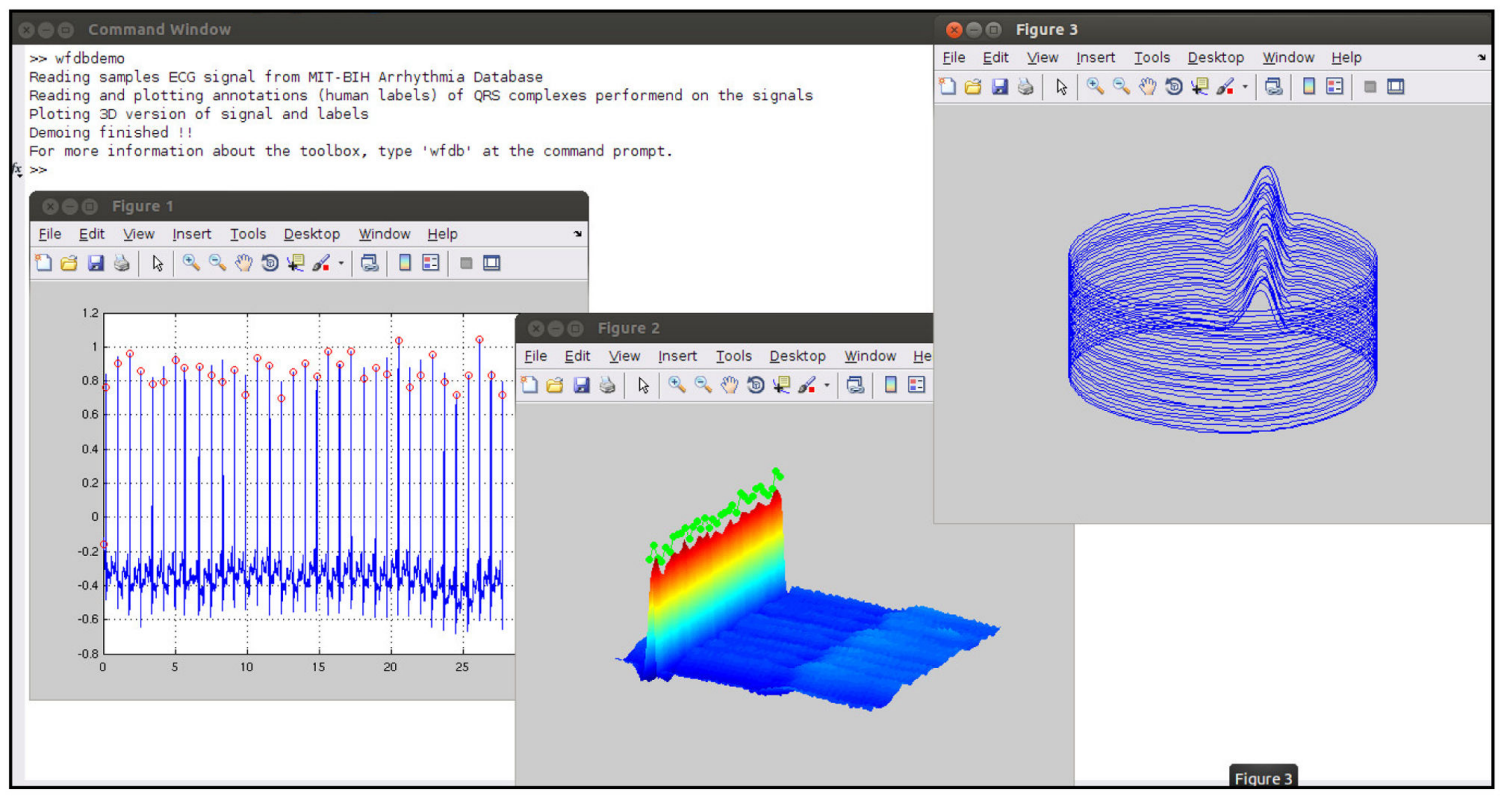
The WFDB Toolbox for MATLAB/Octave integrates PhysioNet’s software and databases with the high precision numerical computation and environment of MATLAB/Octave. Results of the demo script, WFDBDEMO, are shown.

The WaveForm DataBase (WFDB) Toolbox for MATLAB/Octave (see Figure 1) is a collection of over 30 functions and utilities that integrate PhysioNet’s open-source applications and databases with the high-precision numerical computational and graphics environment of MATLAB and Octave. The WFDB Toolbox for MATLAB/Octave is an open-source project supported by PhysioNet [1], with a dedicated support mailing address at: wfdb-matlab-support@physionet.org. A managed community forum for discussions is also available at https://groups.google.com/forum/#!forum/wfdb-app-toolbox.

The WFDB Toolbox for MATLAB and Octave currently provides direct access to over 50 databases in PhysioNet (around 3 TB of data). The function RDSAMP allows users to load PhysioNet waveform data into MATLAB’s or Octave’s workspace; if the database signals are not cached locally, they are automatically fetched from PhysioNet’s servers using HTTP and stored locally for future access. In addition, the function RDANN allows users to load meta data (defined as “annotations” in WFDB terminology). The annotations are generated by medical experts examining the raw waveforms, or by sophisticated published medical algorithms. Examples of the types of annotations available include arrhythmia events, evoked potential epoch markers, epileptic seizure onset, sleep stages, apnea events, and signal quality indication. The PHYSIONETDB function allow users to browse PhysioNet’s databases within MATLAB/Octave. The output argument of PHYSIONETDB, a cell array following the input syntax of RDSAMP, provides a convenient way to process all databases and signals in PhysioNet, using only two ‘for’ loops and RDSAMP.

The WDFB Toolbox also provides functions useful for processing physiological signals. Some of these functions consist of MATLAB/Octave wrappers for open-source C-code functions published in the literature and contributed by user’s to PhysioNet. Among the currently implemented wrappers to popular third-party C-code software are ECGPUWAVE [2–5], EDR [6], MSENTROPY [7–8], and WABP [9]. The WFDB Toolbox also contains a wrapper, BXB, to a function for testing and reporting performance results of cardiac rhythm and ST segment measurement algorithms according to ANSI/AAMI EC38:1998 [10–11]. Thus this library and the databases provide a vital resource for MATLAB and Octave users who want to learn, validate, and compare different biosignal processing algorithms.

### Implementation and architecture

**Figure 2:**
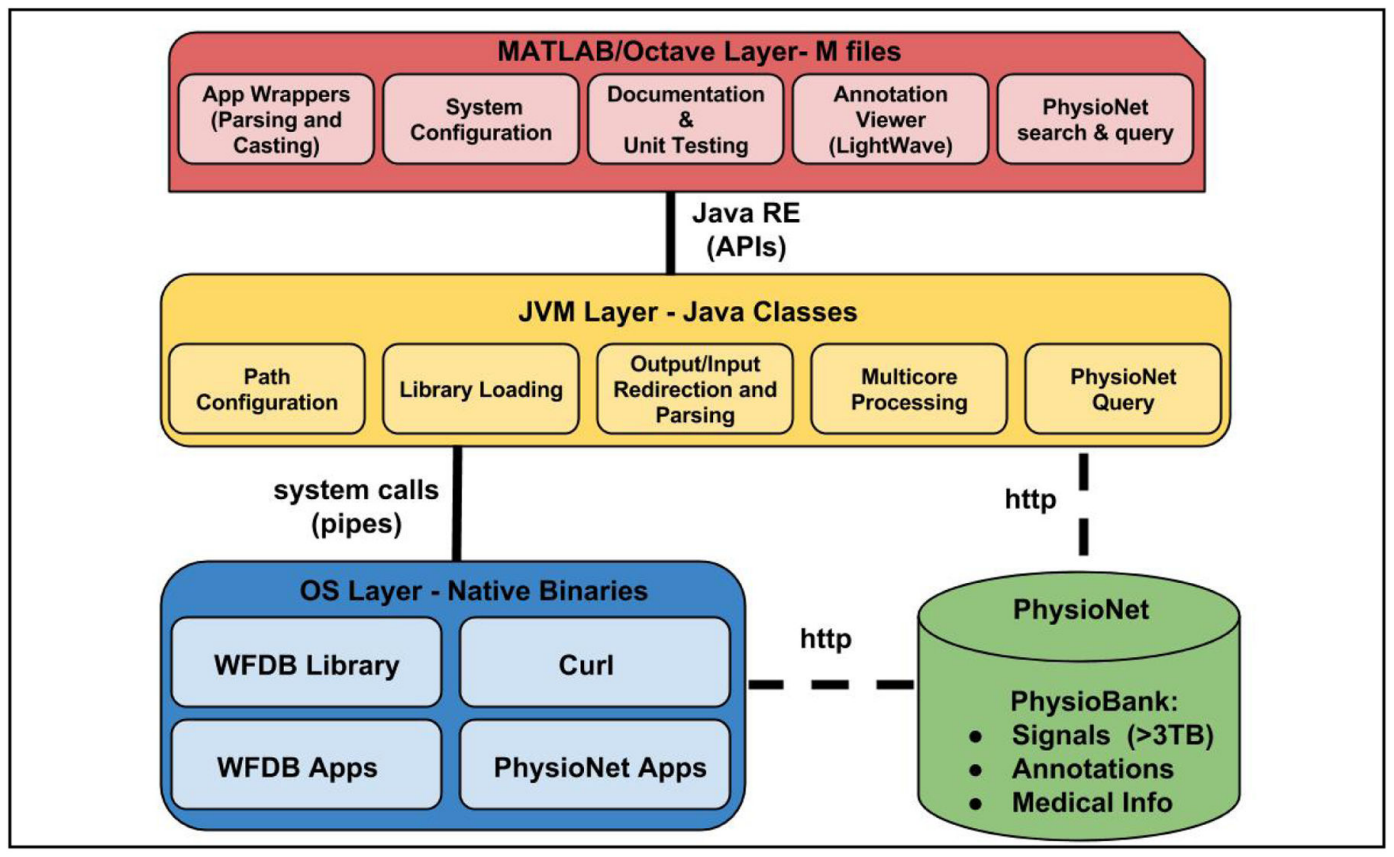
A schematic representation of the structure of WFDB Toolbox for MATLAB/Octave.

**Figure 3:**
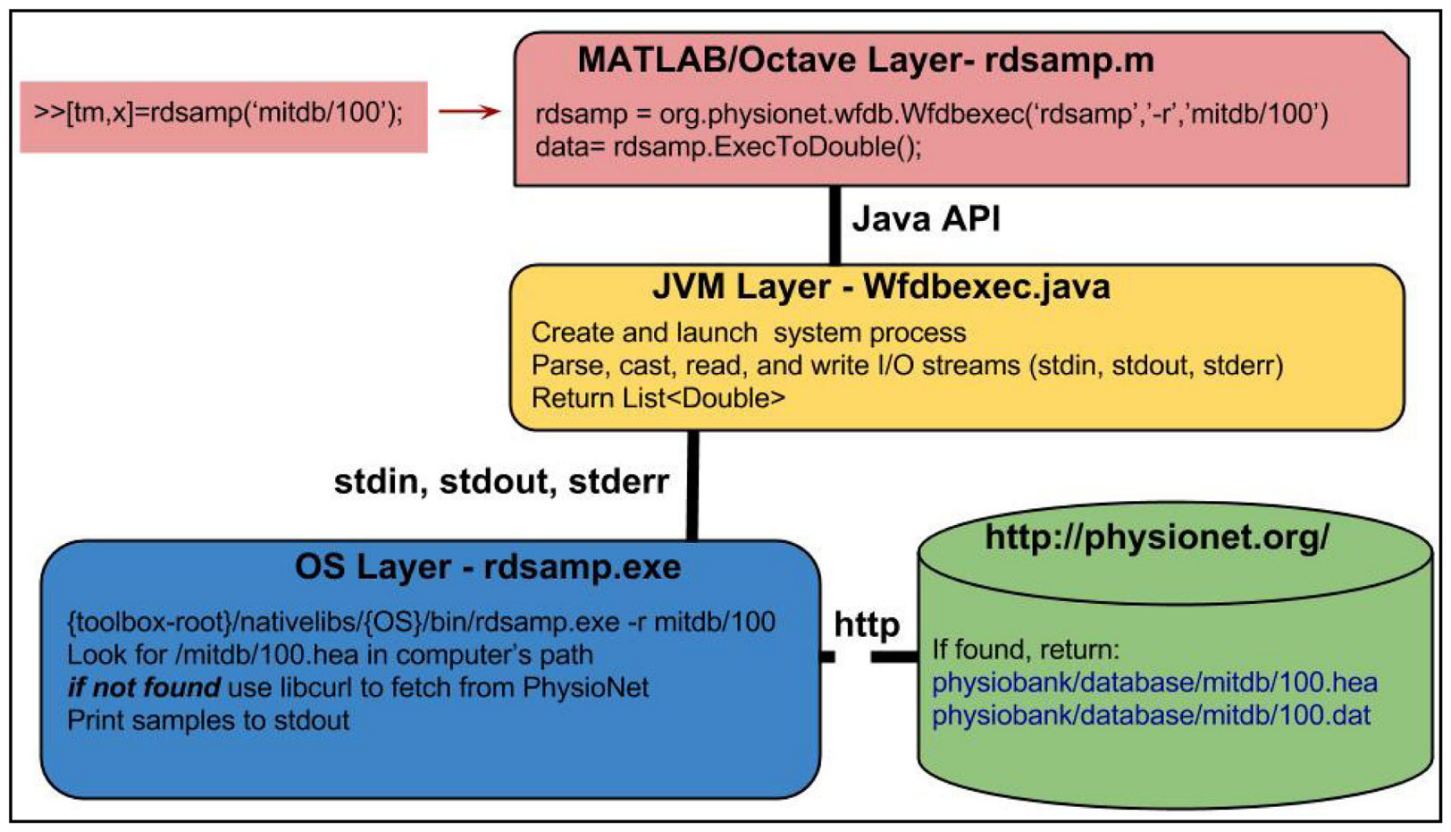
The WFDB Toolbox for MATLAB/Octave integrates PhysioNet’s software and databases with the high precision numerical computation and environment of MATLAB/Octave. Results of the demo script, WFDBDEMO, are shown.

A schematic diagram of the software architecture is shown in Figure 2. The top level of the toolbox consists of HTML documentation files and *.m files. The MATLAB/Octave layer is responsible for interfacing between the user and the toolbox. Additional files not shipped with the toolbox, but available at the code repository, are used for unit testing at the MATLAB/Octave layer. This MATLAB/Octave layer is linked through a Java API (provided either through MATLAB or Octave) to a set of Java classes. These Java classes are responsible for path configuration, library/binary loading, I/O parsing, multi-thread processing, and basic database queries. The Java classes are linked to WFDB native binaries through standard input, output, and error pipes that are created when the JVM performs a system call. An example of this framework in the specific case of reading PhysioNet data using RDSAMP on a Windows environment is shown in Figure 3.

### Quality control

A unit-test framework has been specifically developed for the toolbox, in order to ensure consistent testing in both MATLAB and Octave environments. The goal of the unit tests is to certify a minimum quality prior to public releases. These test suites were individually run on 64-bit versions of GNU Octave 3.6.4, MATLAB 2013a/2013b, Windows 7, Mac OS X 10.9, and Linux Ubuntu 13. These tests ensure that all documented examples work as expected in all the supported environments. In some cases, tests for known issues were also added. These issues are not yet fixed, but have been documented through the repository tracking system. The unit test framework, and the list of known issues and bugs in the code repository are updated and maintained by the PhysioNet developers.

## (2) Availability

### Operating system

The software was tested on 64-bit versions of Linux, Mac OS X 10.9, and Windows 7. The WFDB Toolbox for MATLAB/Octave can be configured to work on other systems, but support is not provided for systems other than those in which unit tests were performed.

### Programming language

Java 1.6, MATLAB 2013a/2013b, C, bash, and Octave 3.6.4 or higher.

### Additional system requirements

N/A

### Dependencies

Libcurl 7.33.0 or higher for accessing PhysioNet databases via a HTTP connection.

### List of contributors

Michael Craig and Daniel J. Scott helped with the development of the first few revisions of the alpha version of the software.

Gari D. Clifford provided valuable guidance on toolbox requirements and features.

Developers who have contributed open-source C code to PhysioNet that is implemented in the WFDB Toolbox for MATLAB/Octave are credited on the help pages of the respective MATLAB wrappers to their software.

An up-to-date list of users who have contribute with bug reports, fixes, testing, or enhancement requests is maintained at the project’s web page in PhysioNet.

### Archive

***Name***
  PhysioNet
***Persistent identifier***
  http://physionet.org/physiotools/matlab/wfdb-app-matlab/
***License***
  GNU GPL v3
***Publisher***
  PhysioNet
***Date published***
  11/02/2014

### Code Repository

***Name***
  Google Code
***Identifier***
  http://code.google.com/p/wfdb-app-toolbox/
***License***
  GNU GPL v3
***Date published***
  11/02/2014

### Language

Java, MATLAB, C, Octave, POSIX

## (3) Reuse potential

This software should be of interest to any user who wishes to do signal processing research with biomedical data. This software can be used as valuable resource for reporting and validating scientific and industry results. PhysioNet encourages the use of this toolbox and its accessible databases for educational purposes as well.
